# Dynamic Road Anomaly Detection: Harnessing Smartphone Accelerometer Data with Incremental Concept Drift Detection and Classification

**DOI:** 10.3390/s24248112

**Published:** 2024-12-19

**Authors:** Imen Ferjani, Suleiman Ali Alsaif

**Affiliations:** Deanship of Preparatory Year and Supporting Studies, Imam Abdulrahman Bin Faisal University, Dammam 31441, Saudi Arabia; saalsaif@iau.edu.sa

**Keywords:** accelerometer sensor, incremental learning, concept drift, road anomalies detection, Multilayer Perceptron

## Abstract

Effective monitoring of road conditions is crucial for ensuring safe and efficient transportation systems. By leveraging the power of crowd-sourced smartphone sensor data, road condition monitoring can be conducted in real-time, providing valuable insights for transportation planners, policymakers, and the general public. Previous studies have primarily focused on the use of pre-trained machine learning models and threshold-based methods for anomaly classification, which may not be suitable for real-world scenarios that require incremental detection and classification. As a result, there is a need for novel approaches that can adapt to changing data environments and perform effective classification without relying on pre-existing training data. This study introduces a novel, real-time road condition monitoring technique harnessing smartphone sensor data, addressing the limitations of pre-trained models that lack adaptability in dynamic environments. A hybrid anomaly detection method, combining unsupervised and supervised learning, is proposed to effectively manage concept drift, demonstrating a significant improvement in accuracy and robustness with a 96% success rate. The findings underscore the potential of incremental learning to enhance model responsiveness and efficiency in distinguishing various road anomalies, offering a promising direction for future transportation safety and resource optimization strategies.

## 1. Introduction

The automatic detection of road anomalies has become increasingly vital in the quest for enhancing road safety and efficiency. In the context of a rapidly urbanizing world, the ability to detect and respond to road conditions like potholes, cracks, and other irregularities is not just a matter of convenience, but a critical necessity to prevent accidents, reduce vehicle damage, and ensure optimal road maintenance.

With the advent of advanced technologies, especially the ubiquity of smartphones, leveraging accelerometer signals has emerged as a promising method for real-time road surface monitoring. These signals, capable of capturing three-dimensional acceleration data, provide a rich source of information about road conditions. However, the dynamic and unpredictable nature of road environments poses unique challenges. The key challenge lies in the concept of “data drift” or “concept drift”—a phenomenon where the statistical properties of data change over time, rendering static machine learning models less effective.

Our study is motivated by the need to address this challenge. Traditional methods, largely relying on pre-trained models, struggle to adapt to new data patterns that emerge as road conditions evolve. This inadequacy can lead to significant issues in safety-critical applications, such as misclassifications or failure to detect new types of anomalies.

While our study focuses on smartphone accelerometer data, the proposed method is potentially applicable to various accelerometer-based data acquisition systems, including vehicle-mounted sensors and dedicated road surface monitoring equipment. The core algorithms for feature extraction, concept drift detection, and classification are agnostic to the specific data source, as long as it provides similar accelerometer readings. However, further research would be needed to validate the method’s performance on these alternative data collection systems.

The primary objective of this research is to develop an innovative approach that employs incremental learning techniques for real-time road anomaly detection using smartphone accelerometer data. This approach aims to overcome the limitations of traditional static models by adapting continuously to concept drifts.

The specific research questions guiding this study are:

- How can we design a machine learning model that dynamically adapts to continuous changes in data patterns for effective road anomaly detection?
- What strategies can be employed to optimize the use of computational and memory resources in processing extensive sensor data in real-time?
- How does the proposed incremental learning model compare in performance and efficiency with traditional static models in the context of real-time road condition monitoring?

The problem we address in this study is twofold. Firstly, there is the technical challenge of designing a system that can adapt to the ever-changing data characteristics inherent in real-world road conditions. This requires the development of a model that not only learns from new data but also retains its effectiveness over time without the need for periodic retraining. Secondly, the system must be efficient in its resource consumption, given the voluminous nature of sensor data collected from smartphones. This efficiency is crucial for practical deployment, especially in scenarios where real-time processing is essential.

It has been extensively studied in recent years how to detect and classify concept drift. The research conducted by Mehta et al. [1] has focused on understanding how changes in the behaviour of data can affect the accuracy of traditional mining techniques. The researchers have shown that Data streams are time-variant in nature, and applying any mining algorithm in a single scan of data is a challenging task. It also points out the open research questions in this field, indicating that further research is needed to improve data stream analysis.

In Souza et al. research [2], the authors contribute to the development of concept drift detection methods for high-speed and high-dimensional data streams, which is essential for real-time data analysis in real-world applications. In addition, the researchers in [3] developed a hybrid method using threshold-based and machine learning approaches for near real-time detection and classification of road surface anomalies using smartphone sensor data. The proposed algorithm has self-adapting and self-updating capabilities to adapt itself to any type of smartphone and the dynamic behaviours of various vehicles and road surface conditions. Other recent research [4,5,6] demonstrate that the use of pre-trained machine learning models for classifying real-time accelerometer signals can achieve high accuracy and efficiency in detecting various road anomalies. However, one limitation of these approaches is that the pre-trained models do not adapt to new features present in the incoming data, which can lead to decreased performance and accuracy over time. This is particularly problematic in scenarios where the data is highly dynamic and subject to frequent changes, such as road anomalies detection or human activity recognition. Failing to update the pre-trained model can result in misclassifications and false alarms, which can have serious consequences in safety-critical applications.

Overall, while pre-trained machine learning models have shown significant promise in classifying real-time accelerometer signals, their lack of adaptability to upcoming features can limit their performance and accuracy over time. The development of techniques for adapting pre-trained models using incremental learning has the potential to address this limitation and improve the reliability and safety of real-time applications.

The cornerstone of our contribution lies in the integration of an incremental learning approach, a novel endeavor in the realm of road anomaly detection using smartphone accelerometer data. Unlike traditional models that operate on static datasets and require periodic retraining, our model is designed to learn continuously and adapt in real-time to evolving data patterns. This ability to dynamically incorporate new data and adjust to concept drift in real-time is a significant advancement over existing methodologies. Furthermore, our approach is meticulously crafted to optimize memory and computational resource usage, which is paramount in processing large volumes of sensor data efficiently. The real-time adaptation to new features without the need for retraining or overwriting previous knowledge positions our model at the forefront of innovation in road safety applications. These features make our system not only more adaptive to the dynamic nature of real-world data but also more efficient and scalable. This approach is particularly relevant in scenarios where road conditions are constantly changing, necessitating a system that can promptly learn and adapt. Overall, our research represents a significant leap forward in the application of machine learning for road anomaly detection, setting a new benchmark for accuracy, efficiency, and adaptability in the field.

Our research introduced significant advancements in the field, including:

- An Innovative Incremental Learning Framework: We present a cutting-edge incremental learning architecture specifically tailored to process streamed accelerometer data. This architecture is designed to dynamically adapt to changes in data patterns, ensuring ongoing relevance in real-time road anomaly detection.
- Real-Time Classification Excellence: Our system excels in the real-time classification of road anomalies, promptly identifying and responding to concept drifts in the data. This means that our solution is highly responsive to changing road conditions, delivering a heightened level of accuracy and safety for road users.
- Adaptive Feature Update Strategy: We have devised a sophisticated feature update strategy that allows us to seamlessly incorporate upcoming features into the existing model without erasing previously acquired knowledge. This approach ensures the model’s adaptability to evolving data patterns while maintaining its wisdom from past experiences.
- Resource Optimization: Our meticulously designed architecture is adept at optimizing the utilization of computational and storage resources, making it exceptionally efficient when processing extensive volumes of sensor data sourced from smartphones. This optimization is critical for achieving real-time performance without compromising the quality of results.

## 2. Methods and Materials

### 2.1. System Modeling

**Figure 1:**
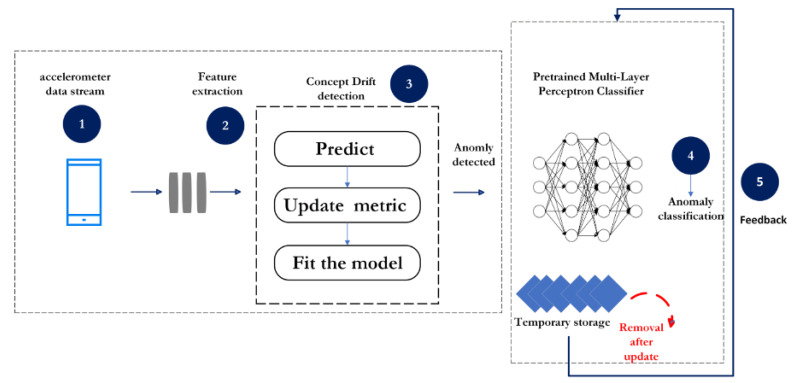
A general flow of incremental machine learning based approach for road anomalies detection. Image credit: ©draw.io.

As illustrated in Figure 1, the proposed system initiates with the streaming of accelerometer signals, which serve as the primary input data. These signals are segmented into discrete time windows, with each window capturing accelerometer readings at specified intervals. Within each window, the system employs a dual-faceted feature extraction strategy as outlined in Table 1. This involves statistical analysis for immediate pattern recognition and a Discrete Wavelet Transform (DWT) to decompose the signal into coefficients that reveal underlying frequency-based characteristics.

Upon the extraction of these features, a concept drift detection algorithm is engaged, which scrutinizes the data for any deviations from the expected distribution, indicative of potential anomalies. When such a drift is detected within a window, suggesting an anomaly, it triggers the classification phase of the system. Here, a pre-trained Multilayer Perceptron (MLP) classifier is tasked with identifying the specific type of anomaly present.

**Figure 2:**
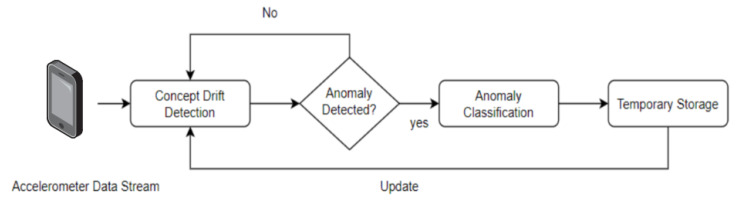
Flowchart outlining the main phases of the machine learning-based road anomaly detection system. Image credit: ©draw.io.

Post-classification, the feedback mechanism comes into play, as depicted in Figure 2. The system updates its internal model with the freshly classified data, refining its detection algorithm in the process. This feedback loop is pivotal, ensuring that the model stays attuned to the dynamic data distributions that characterize real-world environments.

In practice, this continuous cycle of detection, classification, and updating enables the system to maintain high levels of accuracy and adaptability. It evolves over time, learning from new data, which is particularly advantageous for applications that demand real-time processing and immediate responsiveness to changing road conditions.

Figure 1 and Figure 2 provide a visual articulation of this sophisticated, iterative process, highlighting the main phases of our system: data input, feature extraction, anomaly detection and classification, followed by the critical step of updating the model through a temporary storage mechanism that ensures the system’s evolution and relevancy.

### 2.2. Data Stream

**Figure 3:**
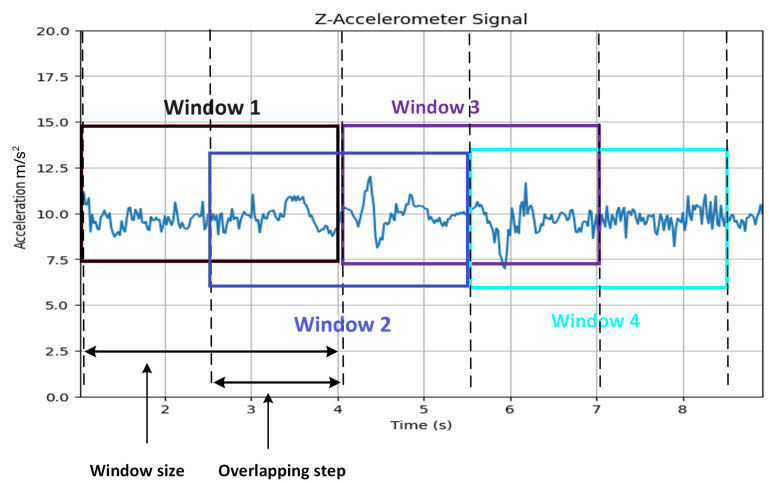
Accelerometer Signal Processing Using Overlapping Sliding Windows. The z-axis accelerometer data is shown with four colored boxes representing distinct sliding windows: Each window overlaps by half its size with the adjacent windows, ensuring continuous and sensitive analysis of the signal for road anomaly detection.

Figure 3 provided demonstrates the use of a sliding window technique in the real-time streaming and processing of accelerometer data, which is the foundational step in our approach. The fixed size of the sliding window determines how many accelerometer readings are captured within each window, with the size itself being contingent upon the specific requirements of the application and the sampling rate of the accelerometer. For instance, with a window size set at 1 s and a sampling rate of 50 Hz, each window will encompass 50 accelerometer readings. Moreover, this windowing strategy involves an overlap of half the window size, meaning that the inception of each new window shares half its data with the end of the preceding window, thereby enhancing the system’s sensitivity to minute variations in the accelerometer signal.

This overlapping configuration is not only beneficial for the sensitivity of anomaly detection but also simplifies computational demands. Unlike methods that process the entire signal in a batch, this technique handles one window at a time, which is computationally more manageable and apt for real-time applications. The approach’s robustness is also notable with respect to changes in the accelerometer’s sampling rate, ensuring the capture of subtle variations in the signals that could indicate road surface anomalies.

In Figure 3, each colored box—Window 1 (Red), Window 2 (Green), Window 3 (Blue), and Window 4 (Purple)—represents a distinct sliding window. The subsequent windows proceed in a staggered fashion, each shifted by the overlapping step, ensuring that no part of the signal is overlooked, particularly at the junctures between windows where anomalies may be most likely to occur. This method ensures continuous monitoring and analysis of the Z-axis accelerometer data, a critical aspect for the real-time detection and classification of road anomalies within the dynamic environment of road surface monitoring.

### 2.3. Accelerometer Configuration and Data Collection

The accelerometer in the smartphone is typically oriented with the Z-axis perpendicular to the phone’s screen, the Y-axis along the phone’s length, and the X-axis along its width. In our setup, the phone was mounted on the vehicle’s dashboard with its screen facing up, ensuring that the Z-axis captures vertical movements most relevant to road anomalies.

While data from all three axes were collected, our analysis focuses primarily on the Z-axis due to its direct correlation with road surface variations. The X and Y axes, which capture lateral and longitudinal movements respectively, were found to be less informative for detecting vertical road anomalies in our preliminary analysis.

The accelerometer used in our study has a sampling rate of 50 Hz and a measurement range of ±2 g, providing sufficient resolution for detecting road anomalies.

### 2.4. Feature Extraction

In the feature extraction phase, our approach meticulously harnesses both statistical and wavelet-based attributes from the data captured within each sliding window. Drawing from the principles outlined in seminal works by Radu et al. [7] and Shannon [8], we derive statistical features that offer insights into the central tendency, dispersion, and shape of the accelerometer signal distribution. These include the mean, which provides a measure of the central value of the signal, and the variance and standard deviation, which reflect the signal’s variability. The coefficient of variation stands as a normalized measure of dispersion, offering comparability across different scales, while the median offers robustness against outliers, and the range provides a simple yet effective measure of signal extent. The integral of the squared signal and the Root Mean Square (RMS) are particularly telling for energy-based anomaly detection, as they relate directly to the power content of the signal.

Wavelet-based features, on the other hand, are derived following the theoretical framework established by Mallat [9], allowing us to delve into the frequency domain characteristics of the signal. By employing Discrete Wavelet Transforms (DWTs), we decompose the signal into its approximate and detailed components, effectively capturing information across various frequency bands. This multilevel decomposition facilitates the extraction of wavelet coefficients at levels 1 to 5, each serving as a proxy for different spectral bands of the accelerometer data. These coefficients are crucial in identifying anomalies that may manifest as transient frequency components not easily discernible in the time domain.

Our feature set, comprising 9 statistical and 15 wavelet features, was identified as the optimal combination through a rigorous sensitivity study by Ferjani et al. [10]. This study compared the effectiveness of various feature sets in the context of road anomaly detection using accelerometer data. The result was a compelling 94 percent accuracy rate, surpassing other feature combinations, including solely time-domain, frequency-domain, and their hybrid counterparts. This robust performance lends credence to our chosen methodology and the dual nature of the feature set, capturing both the temporal and spectral essence of the data.

The concatenation of these 24 features into a feature vector forms a comprehensive representation of each sliding window. This vector becomes the input for a machine learning classifier, which then leverages this rich, multidimensional insight to accurately detect and classify road anomalies in real-time. The efficacy of this approach is further substantiated by the summary Table 1, which details the 9 statistical and 15 wavelet features, offering a clear overview of the multifaceted nature of the data underpinning the model’s decision-making process.

By incorporating both the statistical and wavelet-based features, we ensure a holistic analysis of the accelerometer signals, capturing anomalies that may be evident in either or both domains. This approach not only enhances the detection accuracy but also equips the classifier with a nuanced understanding of the signal characteristics, thereby ensuring a robust real-time response to road conditions.

### 2.5. Mathematical Formulation

Let $X=\{x_{1},x_{2},\ldots ,x_{n}\}$ be the stream of accelerometer data, where each $x_{i}\in R$ represents a 1-dimensional accelerometer reading. We define a sliding window $W_{t}$ of size *w* at time *t* as:

(1) $$W_{t}=\left\{x_{t-w+1},x_{t-w+2},\ldots ,x_{t}\right\}$$

For each window, we extract a feature vector $f_{t}\in R^{d}$, where *d* is the number of features. The feature extraction process can be represented as a function *F*:

(2) $$f_{t}=F\left(W_{t}\right)$$

Our concept drift detection algorithm uses a Softmax Regression model. Given *K* classes, the probability of a sample belonging to class *j* is:

(3) $$P\left(Y=j|X=x\right)=\frac{e^{\theta_{j}^{T}x}}{\sum_{k=1}^{K}e^{\theta_{k}^{T}x}}$$

where $\theta_{j}$ are the model parameters for class *j*.

The incremental learning process updates the model parameters θ based on new data:

(4) $$\theta_{t+1}=\theta_{t}-\eta \nabla L\left(\theta_{t},x_{t},y_{t}\right)$$

where η is the learning rate, and *L* is the loss function.

### 2.6. Concept Drift Detection

The third step in the proposed approach is to detect concept drift, a phenomenon in which the statistical and wavelet properties of the incoming data change over time, which leads to a degradation in the performance of a machine learning model that is trained on a stationary dataset. Our concept drift detection algorithm utilizes the Softmax Regression model introduced in Equation (3). The model is initialized with a learning rate η of 0.01 and uses stochastic gradient descent for optimization. It processes each incoming sliding window feature vector f_t, extracted as per Equation (2), updating its parameters θ after each batch of 100 samples using the incremental learning process described in Equation (4).

Concept drift is detected by monitoring the model’s prediction error rate, calculated as the difference between the predicted probabilities (given by Equation (3)) and the true labels. If this error rate exceeds a predefined threshold (set at 0.2 in our experiments) over a sliding window of 1000 samples, as defined in Equation (1), it signals a concept drift. Upon detection of concept drift, the model’s learning rate η in Equation (4) is temporarily increased to 0.1 for the next 500 samples to quickly adapt to the new concept. This adaptive learning rate allows the model to rapidly adjust to changes in the underlying data distribution. When the model detects an anomaly in an incoming window, the window is flagged as anomalous and sent to the classification model for further analysis.

Detection of concept drift is crucial for maintaining the accuracy of the proposed approach in detecting road anomalies, as it allows the model to adapt to changes in incoming data over time and maintain high classification quality.

### 2.7. Anomaly Classification

When an anomaly is detected by the concept drift detection module, the fourth step in the proposed approach is anomaly classification. A pre-trained Multilayer Perceptron (MLP) model is used to determine the type of anomaly present when an anomaly is detected. A large dataset of labeled accelerometer data is used in this approach, which includes a variety of road anomalies such as potholes, speed bumps, and road humps. The model is trained by mapping the input data to the corresponding output labels using a supervised learning approach. When trained, the MLP model is capable of classifying accelerometer data into the different anomaly classes with high accuracy. Model inputs are the feature vectors extracted from the incoming window, and the MLP model is implemented using a Python library (Keras). The model output is a probability distribution over the different anomaly classes. This indicates the likelihood of each class being present in the current window. The proposed method provides a more detailed and insightful analysis of road conditions by performing anomaly classification on incoming data, which can be used for a variety of applications including road maintenance and safety monitoring.

### 2.8. Temporary Storage

The temporary storage in our anomaly detection model serves as a dynamic repository for recent accelerometer samples. Its role is pivotal in enhancing the model’s adaptability and long-term performance. By enabling the anomaly detector to learn from recent real-world data, the system becomes more robust against novel or evolving road conditions. This ongoing learning process ensures that the model remains effective and reliable, even as the characteristics of the data it analyzes change over time.

During real-time monitoring, the anomaly detector scrutinizes incoming accelerometer readings to detect potential anomalies. Once an anomaly is detected, the relevant sample is passed on to the classifier for determining the type of anomaly. Simultaneously, recent samples, including the one containing the detected anomaly, are stored in the temporary storage.

The data accumulated in the temporary storage is not immediately discarded after classification. Instead, it is retained for a brief period. This stored data is then used to update the anomaly detector. The update involves integrating new information and features from the recent samples into the detector’s learning mechanism. Through this process, the anomaly detector evolves and adapts to new patterns or types of anomalies that may not have been present or considered during its initial training phase. The temporary storage is structured to hold a limited and recent subset of data for updating the anomaly detector. Post-update, the storage is cleared to make space for new incoming data, maintaining a continuous cycle of learning and adaptation. This approach ensures that the system’s memory usage is optimized, focusing only on pertinent data and avoiding resource wastage. The temporary storage in our system retains data for a period of 5 min. This duration was determined through empirical testing, balancing the need for recent data representation against computational resource constraints. Factors considered in determining this optimal value include:

1. The frequency of road anomalies encountered in typical driving scenarios.
2. The rate of concept drift observed in our preliminary studies.
3. The computational resources available on typical smartphone devices.
4. The need to maintain system responsiveness for real-time detection.

This 5-min window allows the system to adapt to gradual changes in road conditions while preventing outdated data from influencing current predictions.

### 2.9. Feedback Loop

The feedback loop is the final step in the proposed approach for ensuring that the concept drift detection module is capable of adapting to changes in the underlying data distribution over time. An output of the anomaly classification module, namely the type of anomaly detected in the current window, is fed back into the concept drift detection algorithm. Through this feedback loop, the concept drift detection algorithm can update its internal model based on new information, improving its accuracy in detecting anomalies in future accelerometer data windows. The update is made in an online fashion. The Softmax Regression algorithm adjusts the probability distribution over the different anomaly classes based on feedback. Detecting certain types of anomalies in incoming data consistently will increase the probability of that class, while decreasing the probability of other classes. In order to detect anomalies accurately, the model must be able to adapt to changes in the underlying data distribution over time. A batch of samples can be used to update the gradients of the Multilayer Perceptron (MLP) as follows:

(5) $$\Delta W_{ij}=-\eta \frac{\partial E}{\partial W_{ij}}$$

where $\Delta w_{ji}$ is the change in weight between the *j*th neuron in the (l−1)th layer and the *i*th neuron in the *l*th layer, η is the learning rate, and $\frac{\partial E}{\partial w_{ji}}$ is the partial derivative of the error function *E* with respect to the weight $w_{ji}$. This can be calculated using the backpropagation algorithm, which recursively computes the error gradients for each layer of the network. The weight update is then applied using the gradient descent algorithm, which adjusts the weights in the direction of the negative gradient to minimize the error function.

This feedback loop involves a cyclical process, in which the updated model is used to classify new incoming accelerometer data windows. Based on the newly acquired information, the anomaly classification module then feeds back the output to the concept drift detection algorithm. As the underlying data distribution changes over time, this iterative approach can continually adapt to maintain a high level of accuracy in detecting road anomalies. An important component of the proposed approach is the feedback loop, which enables the system to adapt to changes in the environment and learn from its mistakes.

### 2.10. Datasets

In order to validate our study, we used two different types of accelerometer measurement datasets which are publicly available [11]: simulated data and real data. In addition to providing valuable insight into the strengths and limitations of our approach, the dataset allowed us to thoroughly evaluate its performance under a wide range of scenarios.

**Figure 4:**
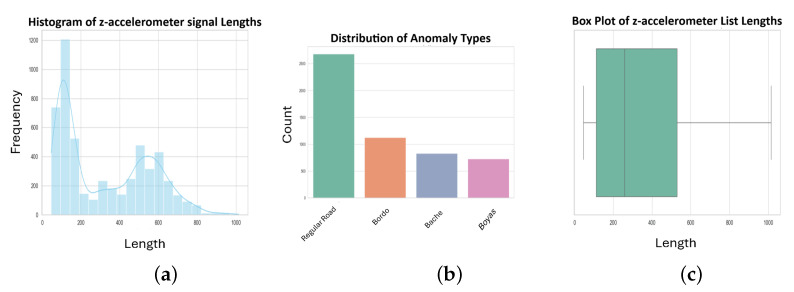
Simulated Data Statistics Visualization.

**Figure 5:**
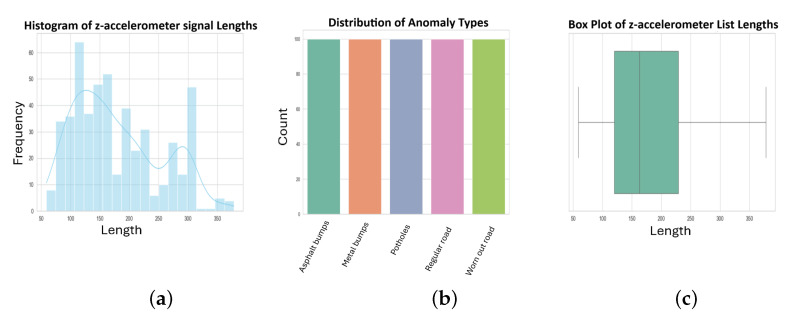
Real Data Statistics Visualization.

Figure 4 and Figure 5 present a comprehensive view of the used dataset statistics. An illustration of the distribution of anomaly types can be seen in the top bar plot, which highlights their respective counts. This box plot and histogram provide insight into the distribution of accelerometer readings’ lengths, revealing the dataset’s structure. By analyzing and modeling these visualizations, we can better understand the dataset’s characteristics.

#### 2.10.1. Simulated Data

The simulated dataset (DB1) was generated using the Pothole Lab web platform [11]. We created virtual roads, each containing various combinations of road anomalies and normal road sections. The roads were classified based on their length and number of anomalies. This approach allowed us to test our system on a wide range of controlled scenarios. As shown in Figure 4, the distribution of anomaly types in DB1 is as follows: ‘Regular Road’ (normal road sections) being the most frequent, followed by ‘Bordo’ (Asphalt speed bumps), ‘Baches’ (potholes), and ‘Boyas’ (Metal speed bumps). The histogram and box plot in Figure 4 illustrate the distribution of z-accelerometer signal lengths, showing a multimodal distribution with most signals falling between 0 and 1000 units in length. There is a detailed description of every virtual road generated in Table 2, including its length, anomalies, and their types. Our approach was tested against a training set and a testing set to ensure it was reliable. Each heuristic’s parameters were calibrated using the training set, aiming for the best values. A key aspect of the usability of any approach is reducing the amount of data needed for calibration. Thus, the training components of the dataset were intentionally made shorter than their testing counterparts.

Additionally, the simulated dataset we used for validation is publicly available, allowing other researchers to compare their results with ours.

#### 2.10.2. Real Data

The real dataset (DB2) utilized in this study was obtained from [12], in which data collection was conducted using 12 vehicles of various makes and models. Accelerometer signals were recorded under diverse driving conditions to ensure robustness and variety in the data. Vehicles traveled at speeds ranging from 20 to 60 km/h on urban and suburban roads with various profiles, including smooth highways, residential streets, and roads with known anomalies. The smartphones used for data collection were securely mounted on the vehicle’s dashboard using standard phone holders, ensuring consistent orientation throughout the process. Accelerometer samples were collected only from the z-axis at a sampling rate of 50 Hz, as this axis is most relevant for detecting vertical road anomalies. In total, more than 130 km were traveled to capture 500 events, which were classified into five distinct categories: metal bumps, worn-out road, potholes, asphalt bumps, and regular road. The dataset’s details, including the distribution of anomalies used, are presented in Table 3. This dataset was selected to validate the proposed approach’s performance in real-world scenarios, as it contains a diverse range of road anomalies.

### 2.11. Model Evaluation Parameters

To ensure a robust and comprehensive evaluation of our proposed approach, we employed a dual validation strategy:

1. 10-fold Cross-validation: We utilized this method to assess the overall performance and generalizability of our models. For each fold, the dataset was divided into 10 equal parts, with 9 parts used for training and 1 part for testing. This process was repeated 10 times, with each part serving as the test set once. The performance metrics were then averaged across all folds.
2. 70-30 Split: In addition to cross-validation, we also performed a fixed 70-30 split of our data, where 70% was used for training and 30% for testing. This split provided a consistent test set for final evaluations and direct comparisons between different models and experiments.

For both validation methods, we evaluated the performance using four key metrics:

1. Accuracy: The proportion of correctly identified anomalies over the total number of instances in the dataset.
  $$\mathrm{Accuracy}=\frac{\mathrm{TP}+\mathrm{TN}}{\mathrm{TP}+\mathrm{TN}+\mathrm{FP}+\mathrm{FN}}$$
2. Precision: The proportion of true positives (correctly detected anomalies) over the total number of predicted anomalies.
  $$\mathrm{Precision}=\frac{\mathrm{TP}}{\mathrm{TP}+\mathrm{FP}}$$
3. Recall: The proportion of true positives over the total number of actual anomalies in the dataset.
  $$\mathrm{Recall}=\frac{\mathrm{TP}}{\mathrm{TP}+\mathrm{FN}}$$
4. F1-score: The harmonic mean of precision and recall, providing a balanced measure of the model’s performance.
  $$F1-\mathrm{score}=2\cdot \frac{\mathrm{Precision}\cdot \mathrm{Recall}}{\mathrm{Precision}+\mathrm{Recall}}$$
  where TP, TN, FP, and FN represent True Positives, True Negatives, False Positives, and False Negatives respectively. These metrics were computed using scikit-learn’s classification_report function. The evaluation of both the anomaly detection and anomaly classification modules was crucial to ensure the proposed approach’s overall effectiveness in detecting and classifying road anomalies. The concept drift detector, being designed for real-time analysis and adaptation, was evaluated based on its performance across both the cross-validation folds and the 30% test set from the fixed split. This dual evaluation approach allows us to benefit from the statistical rigor of cross-validation while also maintaining a consistent test set for final evaluations and comparisons, providing a comprehensive assessment of our system’s performance and adaptability.

## 3. Results and Discussion

### 3.1. Experimental Setup

In the experimental setup section, we conducted five types of experiments to evaluate the performance of our proposed system.

- In the first experiment, we provide an analysis of road anomalies by presenting accelerometer signal insights.
- In the second experiment, we compared our incremental learning system to a baseline approach using the 70-30 split for the baseline model and the 10-fold cross-validation for the overall system performance.
- The third experiment aims to assess our system’s capacity to adjust to the evolving data patterns. We quantify the duration it takes for our system to reach stability and attain precise predictions in the aftermath of a concept drift occurrence. Furthermore, we conduct a comparative analysis of the adaptation performance, specifically examining how it compares to traditional batch learning approaches that necessitate complete retraining processes.
- In the fourth experiment, we quantify the computational resources, including CPU and memory, utilised by your system during incremental learning and prediction tasks. Evaluate the system’s efficiency in terms of resource utilization, especially when dealing with substantial data volumes, and compare it to conventional batch learning methods.
- In the fifth experiment, we explored the impact of different classifiers, including Naive Bayes, Linear Regression, and Softmax Regression, on the model’s ability to adapt and learn incrementally. These classifiers were rigorously tested on two diverse datasets.

In order to mitigate the impact of random variations, we conducted multiple trials of the training and testing procedures across all of our experiments, and we reported the average performance of the detector. In our study, we employed distinct training and testing approaches for different components of the model. Specifically, for the baseline model, we allocated 30% of the data for testing purposes, while the remaining 70% was used for training. This division was aimed at ensuring a comprehensive evaluation of the model’s capabilities. In contrast, for the Multilayer Perceptron (MLP) classifier, a different approach was adopted, with 30% of the data being designated for training. Notably, our concept drift detector operates without the need for traditional training, as it is designed to analyze data in real-time, thereby adapting continuously to evolving patterns without pre-learned data. This methodological distinction underlines the innovative aspects of our model, particularly in its ability to respond dynamically to changing road conditions. In this section, we will refer to the simulated data as ‘DB1’ and the real data as ‘DB2’ for clarity and ease of reference. In-depth details of the experimental setup can be found in Table 4, which provides a comprehensive overview of the variables, conditions, and equipment utilized in the study.

### 3.2. Analysis of Road Anomalies: Accelerometer Signal Insights

In these experiments, we aimed to gain deeper insights into the dynamics of road anomalies and the concept drift in accelerometer signals. These experiments provide a detailed understanding of how different types of road anomalies affect the accelerometer data distribution and highlight the concept drift occurring during these events.

#### 3.2.1. Analysis of Road Anomalies: Accelerometer Signal Insights

**Figure 6:**
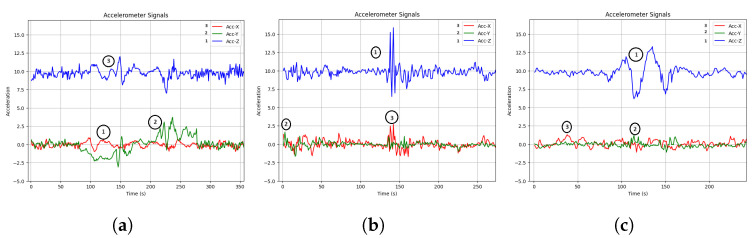
The different accelerometer axis (X, Y and Z) representation during metal bumps (**a**), pothole (**b**) and speed bumps (**c**) anomalies.

In the Figure 6, we delved into the accelerometer signals (X, Y, and Z axes) to analyze their behavior when three specific road anomalies—metal bumps, potholes, and speed bumps—occurred. Notably, we observed that the Z-axis of the accelerometer signal displayed significant variation at the beginning of these anomalies, while the X and Y axes exhibited less pronounced changes. This indicates that the Z-axis is highly sensitive to these specific anomalies, reflecting substantial vertical acceleration.

Moreover, we found that these anomalies have varying durations, with the Z-axis variation occurring primarily at the onset of each anomaly. These findings have practical implications for anomaly detection systems. Sensors or models tuned to monitor Z-axis variations during the initiation of anomalies may be particularly effective in detecting and responding to different types of road surface irregularities.

#### 3.2.2. Assessment of Concept Drift in Accelerometer Data

**Figure 7:**
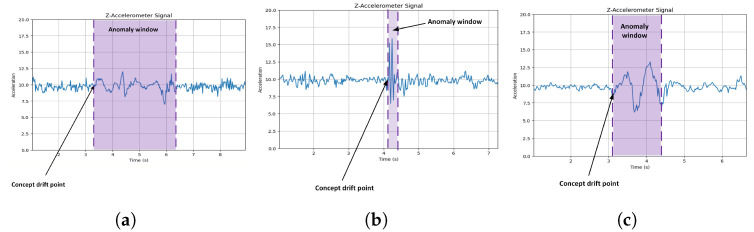
The different accelerometer Z-axis representation during metal bumps (**a**), pothole (**b**) and speed bumps (**c**) anomalies.

In the Figure 7, we investigated concept drift in accelerometer data, particularly focusing on how it manifests during road anomalies. Concept drift refers to changes in data distribution over time, and it’s especially relevant in dynamic environments like road conditions. Our results indicate that concept drift often coincides with the onset of anomalies, causing shifts in the accelerometer data distribution.

Analyzing two different datasets, we observed concept drift in the form of changes in the Z-axis accelerometer data. While the concept drift was more prominent at the beginning of anomalies, its extent and nature varied between datasets. This suggests that different types of road anomalies or distinct road conditions can lead to varying degrees of concept drift.

These experiments have profound implications for real-world applications. By understanding how concept drift occurs during different road anomalies and which accelerometer axes are most affected, we can enhance the design of anomaly detection systems and predictive maintenance strategies. Tuning sensors or models to respond to Z-axis variations at the onset of anomalies may enable more accurate and timely responses to changing road conditions.

In summary, the combined results of these experiments shed light on the intricacies of road anomalies and the concept drift they induce in accelerometer data. This knowledge can contribute to the development of more efficient and robust systems for monitoring and responding to challenging road conditions, ultimately improving road safety and vehicle maintenance practices.

### 3.3. Comparative Analysis of Incremental and Batch Learning Systems

In Table 5, for the batch model, on simulated data, it achieved a high accuracy of 97%, indicating that it correctly classified a significant portion of the data. On real data, the batch model’s accuracy dropped to 79%. This reduction suggests that the model may not generalize well to real-world scenarios. In contrast, the incremental model performed slightly worse on both simulated and real data with accuracies of 96% and 83%, respectively. While the incremental model maintains competitive performance on the simulated data, it is essential to note that it still outperforms the batch model on real data, highlighting its adaptability to real-world conditions. The precision metric measures the proportion of true positive predictions out of all positive predictions made by the model. Both the batch and incremental models show similar precision scores on both simulated and real data. The Recall metric quantifies the ability of the model to identify all relevant instances in the dataset. For both simulated and real data, the batch model and incremental model demonstrate comparable recall scores. The F1-score is the harmonic mean of precision and recall and provides a balance between the two metrics. On simulated data, the batch model achieves an impressive F1-score of 97%. This suggests that it not only identifies anomalies but does so with high precision. The incremental model maintains a similar F1-score on simulated data, implying that it preserves the trade-off between precision and recall effectively.

The comparison of the batch and incremental models on both simulated and real data provides valuable insights into their performance and adaptability. On simulated data, the batch model demonstrates remarkable accuracy and precision, making it highly effective at identifying anomalies. However, its performance declines significantly when applied to real-world data, underscoring the challenges of real-world applications. In contrast, the incremental model, while achieving slightly lower accuracy, exhibits notable adaptability by maintaining competitive performance on real data. This adaptability is a significant advantage, as it indicates the model’s capacity to handle changing data distributions and unseen anomalies over time. Both models strike a balance between precision and recall, indicating their ability to correctly identify anomalies while minimizing false alarms. These results suggest that the incremental model shows promise in real-world applications where data dynamics and shifts are common, highlighting its potential for further development and refinement. Further research and optimization efforts could enhance the models’ performance, making them even more effective in detecting road anomalies.

### 3.4. Adaptation Performance Comparison

**Figure 8:**
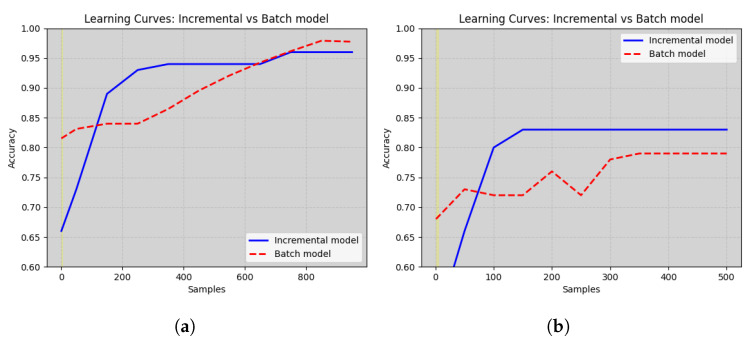
The learning curves of Incremental model and Batch model on simulated data (**a**) and real data (**b**).

In Figure 8, we present a detailed comparison of adaptation performance between our incremental system (represented by the continuous line) and the baseline model (shown in dashed line). Both models are evaluated over increments in time, where each increment corresponds to a concept drift event. The y-axis of the chart measures the accuracy of the models in percentage, while the x-axis indicates time increments. As we observe the figure, several notable insights emerge.

- Baseline Model: At the initial time increment, the baseline model shows an accuracy of 81% and 68% on DB1 and DB2 respectively. Over subsequent time increments, it adapts gradually to concept drifts, displaying a modest increase in accuracy.
- Incremental System: The incremental system, in contrast, starts with an accuracy of 83% and 66% at the initial time increment. As time progresses, the incremental system excels at adaptation. It quickly adjusts to new concepts, leading to a rapid increase in accuracy. The system continues to improve and stabilize, achieving an impressive accuracy of 96% and 83% by the end of the observation period.

This chart vividly illustrates the striking contrast between the incremental system’s dynamic adaptation and the baseline model’s more sluggish response to concept drift events. The data points indicate that our incremental system achieves significantly higher accuracy levels in less time. In summary, the adaptation performance chart provides compelling evidence of the superiority of our incremental system in handling dynamic data environments. It suggests that our model stabilizes quickly and effectively, thus offering more reliable and accurate predictions in the face of evolving data concepts. This chart reinforces the practical significance of our proposed model and its potential benefits in real-world applications.

### 3.5. Computational Efficiency Assessment

**Figure 9:**
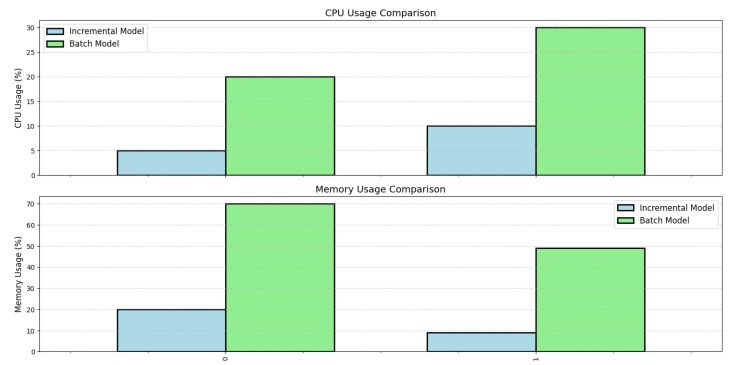
Comparison of CPU and Memory Usage: Resource Efficiency Evaluation of Incremental and Batch Models on Simulated (1) and Real (2) data.

We evaluated the resource utilization of our system during incremental learning and prediction tasks, focusing on CPU and memory usage across two different datasets (Figure 9). Measurements were taken using Python’s psutil library, with each experiment repeated 10 times to ensure reliability. CPU usage was measured as the percentage of total available CPU capacity utilized by our models. The incremental model showed low CPU utilization, with 5% and 10% usage on the first and second datasets respectively. In contrast, the batch model consumed 20% and 30% of CPU capacity on the same datasets. These percentages represent the average CPU utilization over the entire processing time for each dataset. Memory consumption was calculated as the percentage of total system RAM used by our models during execution. The incremental model used 20% of available RAM on the first dataset and 9% on the second, while the batch model required 70% and 49% respectively. These figures represent peak memory usage during model execution. The results consistently demonstrated that our incremental model is more resource-efficient than conventional batch learning methods, both in terms of CPU utilization and memory consumption. This efficiency is particularly advantageous for applications with limited computational resources or when processing large volumes of data in real-time, making our incremental model an optimal choice for scenarios where resource optimization is crucial.

### 3.6. Performance Evaluation of Incremental Learning Models with Classifier Comparison

In this experiment, we conducted an in-depth evaluation of the incremental model developed using the river library. The model was subjected to different classifiers, including Naive Bayes, Linear Regression, and Softmax Regression, and tested on two distinct datasets, namely DB1 and DB2. The evaluation was based on key performance metrics, specifically accuracy, precision, recall, and F1-score, which are essential for assessing the model’s effectiveness in making classifications (Table 6). For Table 6, the same metrics were calculated for each classifier using 10-fold cross-validation. The reported values are the mean scores across all folds.

For DB1, the results showed that Naive Bayes achieved an accuracy of 0.65, a precision of 0.56, a recall of 0.55, and an F1-score of 0.55. In comparison, Linear Regression demonstrated improved performance with an accuracy of 0.66, a precision of 0.64, a recall of 0.61, and an F1-score of 0.61. However, the most remarkable results were obtained by the Softmax Regression classifier, which achieved an impressive accuracy of 0.96, with equally high precision, recall, and an exceptional F1-score of 0.97.

The same trend was observed for DB2, with Softmax Regression outperforming the other classifiers, achieving a similar high level of accuracy, precision, recall, and F1-score.

The analysis of these results indicates that Softmax Regression stands out as the most suitable classifier for this incremental model. It consistently exhibited superior performance across both datasets, demonstrating remarkable accuracy in classifying data points. The high precision, recall, and F1-score further emphasize its capability to make accurate predictions while effectively minimizing false positives and false negatives. The Softmax Regression classifier proves to be robust and versatile, making it the ideal choice for this incremental model in terms of classification accuracy and overall performance.

These findings highlight the importance of selecting the appropriate classifier, as it significantly influences the model’s performance. Softmax Regression’s exceptional results suggest that it is well-suited for the dynamic nature of incremental learning, offering precise and reliable classification on evolving datasets. The high F1-score also underscores its balance between precision and recall, making it an ideal choice for applications where the consequences of false classifications are critical.

### 3.7. Comparison with Literature Works

In the field of road anomaly detection, there has been a consistent pursuit for the development of systems that are not only efficient but also highly reliable. Our study makes a significant contribution to this domain by introducing an innovative approach that harnesses the potential of accelerometer data for the real-time detection of road irregularities. This method is particularly noteworthy when juxtaposed against existing techniques detailed in related works (Table 7). While these established methodologies demonstrate commendable accuracy, they predominantly operate within the confines of batch learning frameworks. Our proposed system, however, distinguishes itself by adopting an incremental learning approach. This design choice endows the system with dynamic responsiveness and the vital ability to adapt quickly to evolving road conditions. Significantly, it also empowers the system to proficiently detect concept drift, a critical aspect often overlooked by traditional methods.

The accuracy of our system, while aligning with that of existing models, is bolstered by its unique adaptability and the efficient utilization of computational resources. These attributes are of paramount importance, especially in scenarios characterized by frequent changes in road conditions or the necessity to process extensive datasets in real-time. Our approach further excels by incorporating online learning capabilities, ensuring that the system continuously hones its predictive accuracy as new data emerges. This ongoing learning process is essential for maintaining relevance and effectiveness in the dynamically evolving landscape of road conditions. Therefore, while the increase in accuracy might be incremental, it is the system’s adaptability and optimized resource usage that truly differentiate our proposed model, heralding it as a notable progression in the field of road anomaly detection.

## 4. Conclusions and Future Work

This study introduces a pioneering approach to road anomaly detection that leverages smartphone accelerometer data through an incremental learning paradigm. Our research addresses the limitations of traditional static models by developing a system that adapts continuously to concept drifts, a crucial capability in the dynamic environment of road conditions.

Our innovative framework, specifically tailored for processing streamed accelerometer data, represents a significant advancement in the field. By implementing a real-time classification system that promptly identifies and responds to concept drifts, we have created a solution that is highly responsive to changing road conditions. This responsiveness translates to enhanced accuracy and safety for road users.

A key feature of our system is its adaptive feature update strategy, which allows for the seamless incorporation of new features into the existing model without erasing previously acquired knowledge. This approach ensures the model’s adaptability to evolving data patterns while maintaining its wisdom from past experiences. Furthermore, our meticulously designed architecture optimizes the utilization of computational and storage resources, making it exceptionally efficient when processing extensive volumes of sensor data sourced from smartphones.

Our evaluation, conducted on both simulated and real datasets, demonstrated the system’s competence with accuracy comparable to traditional batch models. However, the true strength of our system lies in its swift adaptability to changing road conditions and its ability to maintain high performance while optimizing resource usage. This efficiency is particularly advantageous for applications with limited computational resources or when processing large volumes of data in real-time. The implications of this research extend beyond mere technological advancement. By providing a more accurate and efficient method for detecting road anomalies, our system has the potential to significantly improve road safety and maintenance strategies. Transportation planners and policymakers can leverage this technology to make more informed decisions about infrastructure improvements and resource allocation.

Looking ahead, there are several promising avenues for future research. Expanding the scope of detectable road anomalies to include a wider range of environmental factors and unique road conditions could further enhance the system’s versatility. Exploring advanced concept drift detection algorithms and incorporating sophisticated machine learning models, including deep learning constructs, may yield even more accurate results.

Additionally, developing intuitive user interfaces and robust visualization tools for real-time mapping of detected anomalies could greatly enhance the system’s practical applicability. Such tools would enable road maintenance departments to quickly identify problem areas, prioritize repairs, and track the degradation of road conditions over time.

Future studies should also focus on assessing the system’s performance across diverse geographic and environmental contexts to validate its robustness and reliability. Investigating the scalability of the system to manage data from extensive road networks efficiently will be crucial for its widespread adoption.

In conclusion, our research contributes an incremental road anomaly detection system that showcases promising adaptability and resource efficiency. This approach sets a new benchmark for accuracy, efficiency, and adaptability in the field, opening avenues for significant improvements in road safety and maintenance strategies. As we continue to refine and expand this technology, the potential for enhancing transportation infrastructure management in the foreseeable future is vast and exciting.

## Figures and Tables

**Table 1:** Features Summary.

Domain	Feature Name	Description
Time	Mean	Average value of the accelerometer readings
	Integral Square	Sum of the squares of the accelerometer readings
	Variance	Measure of the spread of the accelerometer readings
	Standard Deviation	Square root of the variance
	Median	Middle value of the accelerometer readings
	Range	Difference between the maximum and minimum values of the accelerometer readings
	RMS	Square root of the average of the squares of the accelerometer readings
	Entropy	Measure of the amount of information in the accelerometer signal
Wavelet	Five levels—Daubechies 2	DWT coefficients capturing the high-frequency components at levels from 1 to 5

**Table 2:** The dataset generated with Pothole Lab [11].

Anomaly	Training Set	Testing Set
Potholes	362	155
Metal speed bumps	324	139
Asphalt speed bumps	427	184

**Table 3:** The real dataset used [12].

Anomaly	Training Set	Testing Set
Potholes	70	30
Metal bumps	70	30
Asphalt bumps	70	30
Worn out road	70	30
Regular road	70	30

**Table 4:** Experimental Setup.

Experiment Parameters	Description
Sliding Window Size	30
Feature Extraction Method	Feature extraction was performed using methods detailed in Table 1
Hyperparameters	Softmax Regression classifier for the incremental model; the updating cycle of features is set to 1000 readings
Baseline Model	Multilayer Perceptron (MLP)
Baseline Parameters	hidden_layer_sizes = 100, max_iter = 1000, random_state = 42
Machine Used	Single Nvidia Tesla K80 GPU and 12 GB of RAM

**Table 5:** Comparison of Accuracy Metrics for Incremental and Batch Learning Models in Detecting Road Anomalies. Metrics include Precision, Recall, F1-score, and Overall Accuracy, categorized by Anomaly Types and Datasets.

	Simulated Data				Real Data			
Model	Accuracy	Precision	Recall	F1-Score	Accuracy	Precision	Recall	F1-Score
Batch Model	**97%**	**97%**	**96%**	**97%**	79%	79%	79%	79%
Incremental Model	96%	96%	**96%**	**97%**	**83%**	**82%**	**82%**	**82%**

**Table 6:** Comparison of different online concept drift detectors.

	Simulated Data				Real Data			
Incremental Classifier	Accuracy	Precision	Recall	F1-Score	Accuracy	Precision	Recall	F1-Score
Naive Bayes	0.65	0.56	0.55	0.55	0.36	0.33	0.33	0.35
Linear Regression	0.66	0.64	0.61	0.61	0.57	0.47	0.42	0.42
Softmax Regression	**0.96**	**0.96**	**0.96**	**0.97**	**0.83**	**0.82**	**0.82**	**0.82**

**Table 7:** Accuracy comparison between the best classifier reported in this work and works from the literature.

Author	Detected Road Anomalies	Approach	Accuracy Results
[13]	Potholes	Threshold	92.4%
[14]	Pothole, Bumps, Rough, Smooth uneven	Threshold	85.6%
[15]	potholes, speed bumps, metal humps, rough roads	ANN, Logistic regression	86%
[12]	Potholes, Metal bumps, Asphalt bumps, Regular road, Worn out road	ANN, SVM, DT, RF, NB, KR, KNN	93.8%
[16]	Potholes	Random Forest	95.7%
[17]	speed-breakers, potholes, broken road patches	Decision tree	93%
[18]	Manholes	Support Vector Machine	84.40%
[19]	Potholes, Speed bumps, Curve, Plain	k-Nearest Neighbor	95.55%
[20]	Cobblestones Flatlands, Transits	k-Nearest Neighbor	93.20%
[10]	Potholes, Metal speed bumps, Asphalt speed bumps	MLP, DT, SVM	94%
**This work**	Potholes, Metal speed bumps, Asphalt speed bumps Regular Roads Worn out Road	MLP, Softmax Regression	96%

## Data Availability

The data presented in this study are openly available on GitHub at https://github.com/imenFerjani/Incremental_RoadAnomalies_detection/tree/main/datasets (accessed on 9 September 2024).
